# Graft rejection across solid organ transplants: mechanisms, monitoring, and immunosuppressive therapeutics

**DOI:** 10.3389/fsurg.2026.1762417

**Published:** 2026-04-20

**Authors:** Edward Akosah Danso, Malik Olatunde Oduoye, Williams Chukwuebuka Enuh, Umer Wamiq, Hafsa Shuja, Fnu Sawaira, Hareem Fatima, Sadia Tameez-ud-din

**Affiliations:** 1 Department of Surgery, Korle-Bu Teaching Hospital, Accra, Ghana; 2 Department of Research, The Medical Research Circle (MedReC), Goma, Democratic Republic of Congo; 3 Department of Health Sciences, Hamburg University of Applied Sciences, Hamburg, Germany; 4 Department of Medicine, Jinnah Sindh Medical University, Karachi, Pakistan; 5 Department of Medicine, Khyber Girls Medical College, Peshawar, Pakistan; 6 Dow University of Health Sciences, Karachi, Pakistan; 7 Department of Medicine, Foundation University Medical College, Islamabad, Pakistan

**Keywords:** biological agents, graft rejection, immune tolerance, immunosuppressive therapy, organ transplantation, transplant outcomes

## Abstract

**Background:**

Organ and tissue transplantation has transformed the management of end-stage organ failure, yet graft rejection remains a major barrier. Rejection arises from complex immune mechanisms involving MHC mismatch, T-cell allorecognition, and antibody-mediated injury. Advances in immunosuppressive therapy have improved graft survival, but significant challenges persist.

**Aim:**

This scoping review synthesizes current insights into the immunological basis of graft rejection and evaluates conventional, biologic, and emerging immunosuppressive strategies. Particular attention is given to organ-specific differences and newer fields such as vascularized composite allografts (VCA) and xenotransplantation.

**Methods:**

A systematic literature search was conducted across PubMed, Google Scholar, Cochrane, and ClinicalTrials.gov (updated June 2025) following PRISMA guidelines. Studies addressing mechanisms of rejection, therapeutic innovations, and clinical outcomes in solid organ transplantation were included.

**Results:**

Rejection manifests in distinct forms: hyperacute rejection, though rare due to modern screening, remains catastrophic when pre-existing antibodies are present; acute rejection affects 10%–20% of patients within the first year, driven by both T-cell and antibody-mediated pathways; and chronic rejection, emerging months to years later, leads to progressive fibrosis, vasculopathy, and graft loss across organs. The degree of HLA mismatch consistently emerged as the strongest predictor of long-term survival. Conventional regimens of corticosteroids, calcineurin inhibitors, and antimetabolites remain foundational but are limited by nephrotoxicity, metabolic complications, and infection risk. Biologics such as basiliximab, belatacept, and rituximab have introduced more targeted suppression, while innovative approaches, including regulatory T-cell therapy, tolerogenic dendritic cells, gene-editing strategies, and nanotechnology-based drug delivery, show promise. Despite these advances, long-term therapy is challenged by 20%–70% patient non-adherence, heightened infection risk, and malignancy.

**Conclusion:**

Future strategies must emphasize personalized, biomarker-guided regimens, immune tolerance induction, and AI-driven diagnostics to achieve durable graft acceptance with minimal complications. Integration of consensus frameworks and precision medicine approaches will be essential to improving long-term graft survival and patient health.

## Scope and purpose

This review synthesizes current evidence on the immunological mechanisms underlying graft rejection and the therapeutic strategies used to prevent it across major solid organ transplants. It examines hyperacute, acute, and chronic rejection, emphasizing the roles of HLA mismatch, donor-specific antibodies, ischemia-reperfusion injury, and patient-related factors in shaping outcomes. Conventional immunosuppressive regimens, including corticosteroids, calcineurin inhibitors, and antimetabolites, remain foundational, while biologics such as basiliximab, belatacept, and rituximab provide more targeted suppression. Emerging approaches, including regulatory T-cell therapy, tolerogenic dendritic cells, gene-editing technologies, and nanotechnology-based drug delivery, are evaluated for their potential to improve graft survival and reduce systemic toxicity.

The purpose of this work is to integrate current insights into graft rejection and immunosuppressive therapeutics, identify persistent challenges such as drug toxicity, infection, malignancy, and non-adherence, and outline future directions. By emphasizing personalized medicine, biomarker-guided therapy, immune tolerance induction, and AI-driven diagnostics, this review aims to inform clinical practice and guide research toward precision-based strategies that maximize graft survival while minimizing complications.

## Highlights

- This review synthesizes organ-specific insights into graft rejection and emerging strategies, including personalized immunosuppressive regimens, AI-driven risk prediction, bioengineered grafts, and novel therapeutics.
- Graft rejection remains a major barrier in transplantation, driven by complex immune responses involving T-cell activation, B-cell antibody production, alloantigen recognition, and inflammation.
- Hyperacute rejection occurs within minutes to hours due to pre-existing antibodies, while acute rejection typically develops within the first year; chronic rejection emerges later, leading to progressive fibrosis and vasculopathy.
- The degree of HLA mismatch is consistently the strongest predictor of long-term graft survival.
- Advances in biologics, gene therapy, nanotechnology, and tolerance-based approaches show promise in improving outcomes, though long-term success remains challenged by infection risk, malignancy, and patient non-adherence.

## 1 Introduction

Transplantation refers to replacing nonfunctional organs with healthy counterparts obtained either through living donation (e.g., kidney, partial liver) or deceased donation (following brain death or circulatory death criteria) (1). Organ and tissue transplantation has transformed the management of end-stage organ failure, offering patients improved survival and quality of life. Despite these advances, graft rejection remains a major challenge, often compromising long-term success. Rejection arises from complex immune responses in which the recipient's immune system recognizes the graft as foreign, initiating cascades involving MHC mismatch, T cell allorecognition, and inflammatory pathways (2). Ischemia-reperfusion injury further activates innate immunity, stimulating dendritic cells and triggering adaptive responses (3). Acute rejection, particularly within the first six months, is associated with increased risk of chronic complications. For example, chronic allograft nephropathy is a kidney-specific manifestation, whereas other organs exhibit distinct chronic rejection phenotypes (4).

The evolution of immunosuppressive therapies has been central to improving graft survival. Early regimens with glucocorticoids and azathioprine were limited by high rejection rates and adverse effects (5, 6). The introduction of calcineurin inhibitors such as cyclosporine and tacrolimus marked a breakthrough, substantially reducing rejection events (6). Subsequent incorporation of agents like mycophenolate mofetil and mTOR inhibitors further refined protocols, balancing efficacy with safety (7). More recent experimental approaches, including tegoprubart, a monoclonal antibody designed to minimize thromboembolic complications, have shown encouraging early results (8, 9).

While strategies such as regulatory T cell therapy, tolerogenic dendritic cells, costimulation blockade, donor-derived cell-free DNA (cfDNA), and AI-based diagnostics have been extensively reviewed in recent consensus statements, the distinctive contribution of this study lies in its organ-specific synthesis and evidence-graded clinical implications. By comparing outcomes and monitoring approaches across kidney, liver, heart, lung, pancreas, and vascularized composite allografts, this review highlights practical differences that inform individualized transplant care. The aim is to elaborate on the mechanisms underlying graft rejection, examine the evolution of immunosuppressive therapies, and integrate current findings to outline challenges, therapeutic targets, and future strategies for optimizing regimens while minimizing complications.

## 2 Methods

Searches were conducted in PubMed, Cochrane, Google Scholar, and ClinicalTrials.gov from January 2010 to June 2025 using combinations of keywords (“organ transplantation” AND “graft rejection” OR “hyperacute rejection” OR “calcineurin inhibitors”). Filters: English language, human studies. Duplicate records were removed using EndNote. More comprehensive and structured search strategies for each database are presented in Supplmentary Table 1. Two reviewers independently screened titles/abstracts; disagreements were resolved by a third. Risk of bias was assessed using the Cochrane RoB2 tool for RCTs and the Newcastle-Ottawa scale for observational studies.

**Figure 1:**
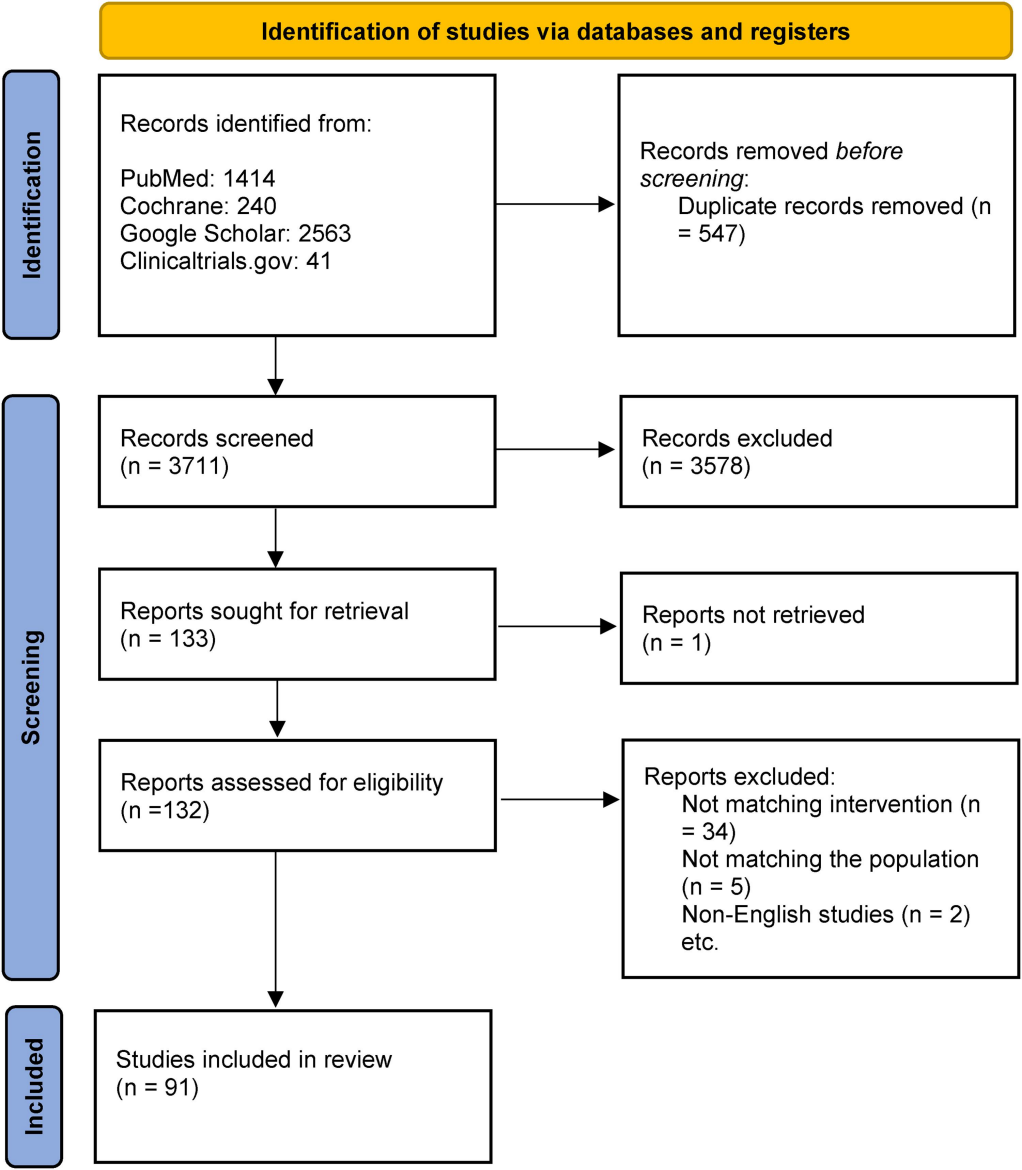
Selection of studies.

The detailed breakdown of study selection is demonstrated in the PRISMA flowchart (Figure 1). Two authors independently screened all identified articles. Titles and abstracts were first reviewed for relevance, and full texts were retrieved when the content matched the scope of this review. Reference lists of included studies were also cross-checked to identify additional eligible publications. Any disagreements were discussed and resolved by consensus with a third author.

### 2.1 Inclusion criteria

Studies were included if they met one or more of the following criteria:

1. Investigations of the immunological mechanisms underlying hyperacute, acute, or chronic rejection.
2. Human studies or relevant large animal models related to organ transplantation (kidney, liver, heart, lung).
3. Reports on conventional and emerging immunosuppressive therapies.
4. Analyses of risk factors, clinical outcomes, or treatment innovations.
5. Original research articles, systematic reviews, meta-analyses, clinical trials, or narrative reviews published in peer-reviewed journals.

### 2.2 Exclusion criteria

Studies were excluded if they met any of the following:

1. Studies that did not fulfill the inclusion criteria outlined above.
2. Studies not published in English.
3. Represented unpublished data or non-peer-reviewed sources.
4. Consisted of editorials, opinion pieces, letters to the editor, or case reports.
5. Originated from manufacturer or marketing webpages, given their limited academic rigor.

## 3 Results

### 3.1 Immunological basis of graft rejection

Transplant rejection is primarily an immune-mediated process in which the recipient's immune system identifies the graft as foreign and mounts a defensive response. This reaction is mediated through both cellular and humoral immunity and is influenced by antigenic differences, primarily the human leukocyte antigen (HLA) system. The graft rejection can be classified into hyperacute, acute, and chronic, based on the differences in onset, mechanisms, and management.

#### 3.1.1 Hyperacute rejection

Hyperacute rejection results from pre-existing antibodies present at reperfusion, historically due to ABO mismatch. In xenotransplantation, cross-species antibodies such as alpha-gal remain the major barrier (10). It occurs as a result of specific recurrent antidonor antibodies against human leukocyte antigen (HLA), ABO, or other antigens (11). These antibodies bind to the vascular endothelium of the transplanted organ, triggering the complement cascade and causing widespread endothelial injury, thrombosis, and immediate and irreversible destruction of the graft (12). Histological examination reveals fibrinoid necrosis, thrombi in capillaries, and neutrophil infiltration (13). Hyperacute rejection is now rare due to rigorous pre-transplant immunological testing, but it remains a critical concern in xenotransplantation or emergency transplant scenarios where prior sensitization is unknown (13).

#### 3.1.2 Acute rejection

Acute rejection can occur at any time after transplantation, though it is most common within the first year. It represents the most frequent and treatable form of graft rejection. The primary mechanisms are T-cell–mediated rejection (TCMR) and antibody-mediated rejection (AMR) (14). In TCMR, recipient T cells recognize donor MHC antigens presented by graft antigen-presenting cells, leading to infiltration, endothelial injury, interstitial inflammation, and tubulitis, particularly in kidney transplants. AMR arises from newly formed donor-specific antibodies that activate the complement cascade, producing microvascular inflammation (13, 15). Early detection and prompt treatment are critical to preserving graft function. Despite advances in immunosuppression, acute rejection still occurs in 10%–20% of patients within the first year (4). Histological features and clinical markers vary by organ. Presenting these side by side helps clarify organ-specific manifestations of acute rejection (Table 1).

**Table 1:** Organ-Specific histology and dysfunction markers in acute rejection.

Organ	Histological findings	Clinical markers of dysfunction
Kidney	Tubulitis, interstitial inflammation	Elevated serum creatinine
Liver	Bile duct injury, portal inflammation, and endothelitis	Elevated liver function tests (ALT, AST, ALP, bilirubin)
Heart	Lymphocyte infiltration with myocyte injury	Reduced ejection fraction, arrhythmias
Lung	Perivascular and bronchiolar lymphocyte infiltration	Decline in FEV1, hypoxemia

#### 3.1.3 Chronic rejection

Chronic rejection is a late complication that may develop months to years after transplantation. Unlike hyperacute or acute rejection, which present abruptly, chronic rejection progresses insidiously, and its immunological mechanisms remain incompletely defined. Both cellular and humoral immune responses contribute, alongside recurrent and persistent inflammation directed against the graft (10). Histologically, chronic rejection is characterized by progressive fibrosis, vascular remodeling, and graft atrophy. Organ-specific manifestations include interstitial fibrosis and tubular atrophy (IFTA) in kidney transplants (16), bronchiolitis obliterans syndrome (BOS) in lung transplants (17), vanishing bile duct syndrome in liver transplants (18), and cardiac allograft vasculopathy (CAV) in heart transplants (19).

The pathogenesis involves chronic low-grade immune injury, often compounded by repeated episodes of subclinical acute rejection (20). Non-immunologic factors also play a role, including ischemia-reperfusion injury (21), drug toxicity, and infections (22). These processes collectively drive vasculopathy, smooth muscle proliferation, and graft arteriosclerosis, ultimately leading to ischemia, dysfunction, and graft loss (23).

### 3.2 Immunological mechanisms of graft rejection

Alloreactive T cells are central to graft rejection, but interactions between T and B cells are critical for generating humoral responses that damage solid organ transplants. The major histocompatibility complex (MHC) on chromosome 6 encodes human leukocyte antigens (HLA), including class I (HLA-A, -B, -C) and class II (HLA-DR, -DP, -DQ) molecules. Class I molecules are expressed on nearly all nucleated cells, while class II molecules are primarily found on antigen-presenting cells but can be induced on other cell types. CD8 T cells recognize peptide/MHC class I complexes, whereas CD4 T cells recognize peptide/MHC class II complexes (24).

**Figure 2:**
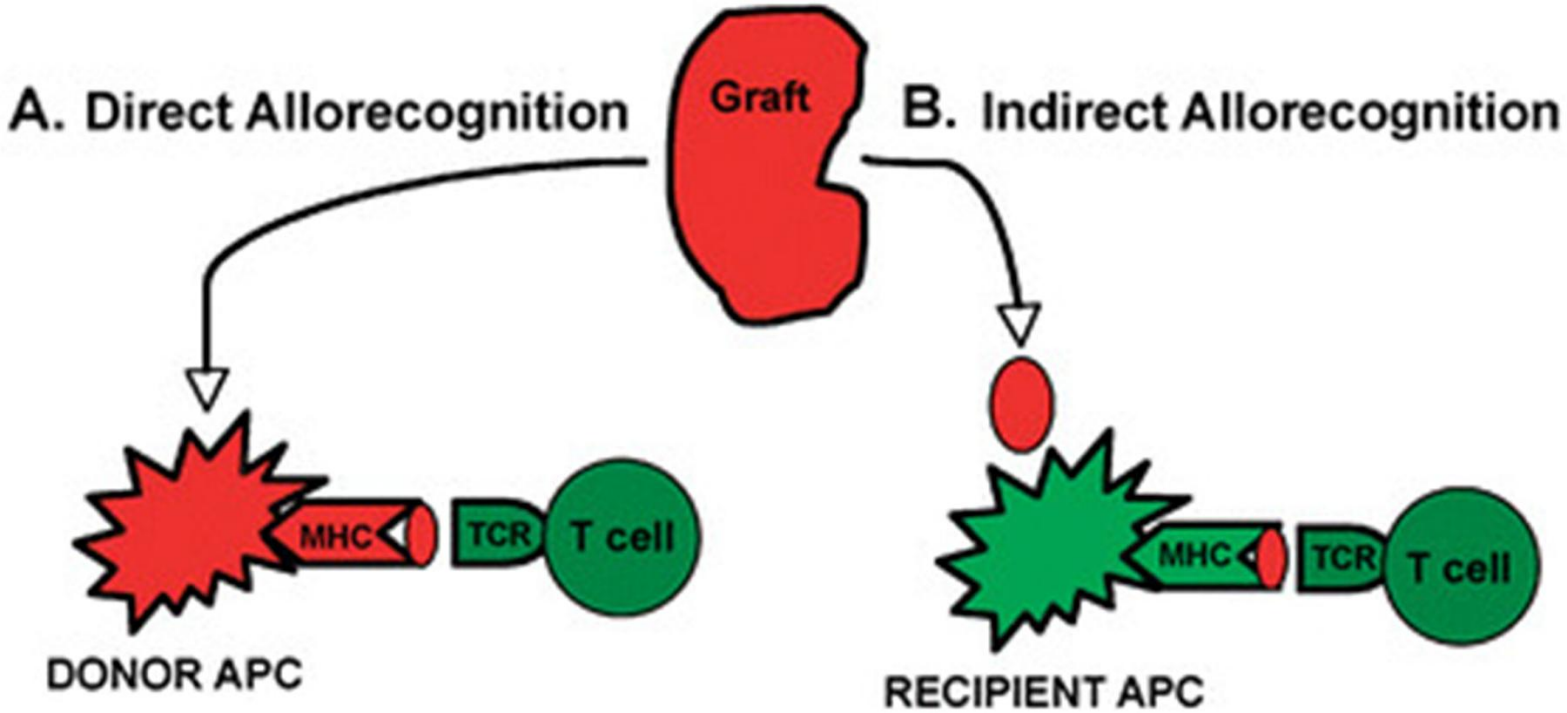
Two distinct pathways of allorecognition. A direct pathway of allorecognition. Dendritic cells migrate from the graft to secondary lymphoid tissues to activate T cells. b Indirect pathway of allorecognition. Graft proteins are processed by recipient dendritic cells and presented to T cells. APC, antigen-presenting cell; TCR, T cell receptor; MHC, major histocompatibility complex (24).

**Figure 3:**
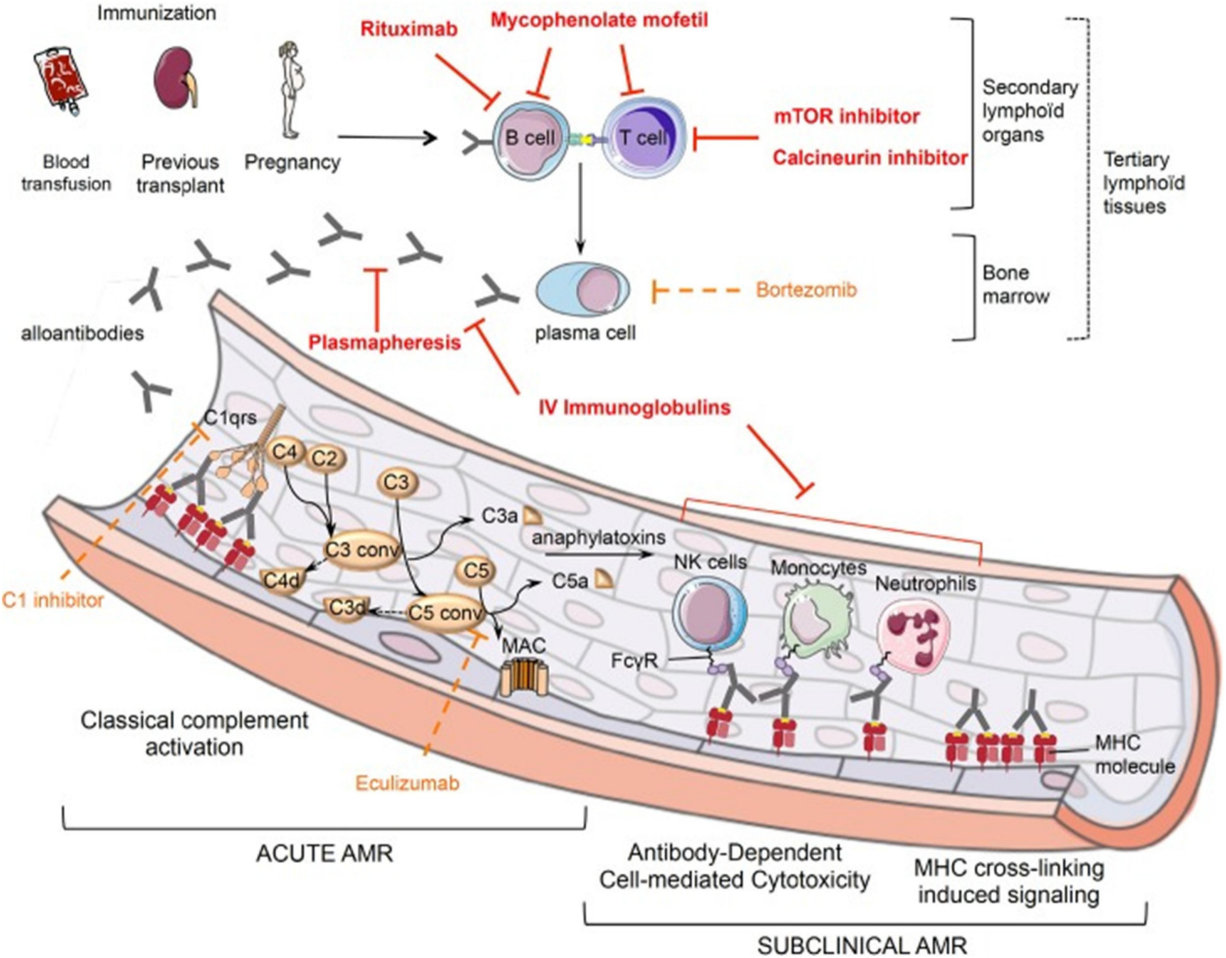
Schematic representation of antibody-mediated rejection pathophysiology (37).

Alloantigen recognition occurs via direct and indirect pathways. In the direct pathway, T cells recognize intact donor MHC molecules, eliciting strong immune responses. In the indirect pathway, donor MHC molecules are processed and presented as peptides by recipient MHC, producing slower but persistent responses (24). Minor histocompatibility antigens also contribute; these non-MHC epitopes can activate both CD4 and CD8 T cells. CD8 T cells (25, 26) and CD4 T cells (27) specific for minor antigens have been isolated in humans and rodents, demonstrating roles in solid organ and corneal rejection, as well as graft-versus-host disease after bone marrow transplantation (28, 29) (Figures 2, 3).

B cells further amplify alloimmunity through multiple mechanisms: differentiating into plasma cells, sustaining humoral immunity, functioning as antigen-presenting cells, supporting tertiary lymphoid organ formation, and producing inflammatory cytokines (30, 31). Plasma cells generate donor-specific antibodies (DSAs), which activate complement cascades, causing vascular injury and promoting both acute and chronic rejection. Beyond complement activation, HLA antibodies can directly injure grafts by binding endothelial cells and engaging Fc receptors on NK cells, macrophages, and neutrophils (32–34). Additional anti-HLA antibodies may target alleles absent in the recipient, as well as minor antigens and autoantigens within the graft (35, 36).

### 3.3 Risk factors of graft rejection

Graft rejection is a multifactorial process defined as the recipient's immune-mediated destruction of transplanted tissue. It is influenced by genetic, immunological, ischemic, and patient-related factors. Human leukocyte antigens (HLA) play a central role in cellular and humoral immune responses after transplantation, particularly in kidney and heart grafts, and remain key determinants of post-transplant outcomes (38). The degree of HLA mismatch is strongly associated with graft survival, with higher allele mismatches correlating with poorer efficacy and increased rejection risk (39). Prior sensitization events, including transfusions, pregnancy, or previous transplants, can lead to donor-specific antibodies that further elevate rejection risk (40, 41). Beyond conventional mismatch assessment, advanced approaches such as Eplet Matching and PIRCHE algorithms provide more precise prediction of rejection risk (42, 43).

Sensitization also raises calculated panel reactive antibody (cPRA) levels, reflecting the burden of donor-specific antibodies (44). Donor-derived cell-free DNA has emerged as an established non-invasive biomarker for detecting allograft injury and rejection. Ischemia-reperfusion injury adds another layer of complexity, with organ-specific differences in tolerance: the heart and lungs are most sensitive to cold ischemia, whereas kidneys demonstrate greater resilience (45, 46). Patient comorbidities, including diabetes, hypertension, and advanced age, further compromise graft function and survival (47, 48). These diverse immunologic and clinical risk factors are summarized in Table 2: Risk Factors Influencing Graft Rejection.

**Table 2:** Risk factors influencing graft rejection.

Risk factor	Mechanism of impact
HLA mismatch	Strong predictor of rejection and graft loss
Sensitizing events (transfusion, pregnancy, prior transplant)	Increase donor-specific antibodies (DSA), raise cPRA
Ischemia-reperfusion injury	Triggers innate immune activation, worsens graft injury
Cold ischemia time	Prolonged times increase the risk of acute rejection
Comorbidities (diabetes, hypertension, age)	Reduce graft function, increase rejection risk

### 3.4 Immunologic and clinical determinants of graft outcomes

A higher rate of mismatched amino acids in donor HLA alleles has been independently associated with increased rejection risk and reduced graft survival (40). Prior exposure to foreign antigens—such as blood transfusions, previous transplants, or pregnancy can lead to donor-specific antibody (DSA) formation, elevating rejection risk. For example, healthy females with one or two pregnancies demonstrate stronger alloimmune responses compared to non-pregnant females (41). DSAs are implicated in both early and late antibody-mediated rejection (AMR), significantly influencing long-term graft survival (42). Accordingly, adequate screening for patient sensitization is essential to assess rejection risk in transplant recipients.

Allograft rejection remains a major obstacle to long-term success. In cardiac transplantation, vascular rejection leading to cardiac allograft vasculopathy accounts for approximately 60% of mortality (43), contributing to chronic dysfunction and graft loss (44). Immune-privileged sites such as the eye and brain exhibit reduced rejection risk compared to highly immunogenic tissues like skin, gut, and bone marrow. Ischemia-reperfusion injury further compromises graft outcomes. Experimental studies in donation after cardiac death (DCD) kidneys demonstrate that both ischemia and reperfusion contribute to delayed graft function and reduced survival (45).

Tolerance to ischemia varies by organ: the heart and lungs withstand only short cold ischemia times, whereas the kidneys show greater resilience. Normothermic machine perfusion is emerging as a preservation strategy to extend ischemia times and mitigate early injury (46). Patient comorbidities, including diabetes, hypertension, and advanced age, also impair graft function and increase risk of renal graft loss (47). Long-term immunosuppression remains necessary to balance drug toxicities against the risk of chronic rejection (48). Beyond these immunologic and clinical factors, long-term outcomes differ substantially by organ type, with graft and patient survival rates summarized in Table 3.

**Table 3:** Graft and patient survival rates by organ.

Organ	1-year survival	3-year survival	5-year survival
Kidney	∼95%	∼90%	∼80%
Liver	∼90%	∼85%	∼75%
Heart	∼85%	∼75%	∼65%
Lung	∼80%	∼65%	∼50%

### 3.5 Advances in immunosuppressive therapy

Immunosuppressive agents have revolutionized transplantation medicine and have been a center of research and development for decades. These drugs prevent organ and tissue rejection by suppressing the immune response by inhibiting the proliferation and function of macrophages, B cells, and T cells. This results in reduced antibody response against the transplanted graft. Immunosuppressive drugs are also used in autoimmune diseases to prevent the generation of an autoimmune response.

#### 3.5.1 Conventional immunosuppressive agents

Conventional immunosuppressants mainly include a combination of corticosteroids, calcineurin inhibitors, and biological agents. These agents work together to reduce T-cell and B-cell activation and proliferation, thus reducing the risk of acute and chronic rejection. Despite their effectiveness, long-term use is associated with adverse effects, mainly nephrotoxicity, high risk of infections, and severe metabolic complications.

##### 3.5.1.1 Corticosteroids

Corticosteroids, such as prednisone, are commonly used immunosuppressive agents to control inflammation and prevent rejection in organ transplant patients and those with autoimmune diseases. They suppress immune responses by inhibiting the activation of T-cells and B-cells, which are key mediators in transplant rejection. While corticosteroids are effective in managing acute transplant rejection, their prolonged use is associated with several side effects, including weight gain, osteoporosis, increased blood sugar, hypertension, and a higher risk of infections (49). These side effects lead to their use in combination with other immunosuppressive drugs, with corticosteroid doses being reduced over time once the immune response is controlled.

##### 3.5.1.2 Calcineurin inhibitors

Calcineurin inhibitors, including cyclosporine and tacrolimus, are essential in preventing organ rejection, particularly in kidney and liver transplants (50, 51). They work by inhibiting calcineurin, an enzyme critical for T-cell activation, thus preventing T-cell proliferation and immune rejection of the transplanted organ (50, 51). Tacrolimus is often preferred over cyclosporine due to its greater potency and a lower incidence of nephrotoxicity. However, both drugs can cause kidney damage, neurotoxicity, and increased susceptibility to infections and cancers, making careful monitoring essential (50, 51). These medications are crucial for long-term transplant success but require dose adjustments based on drug levels to minimize adverse effects.

##### 3.5.1.3 Antimetabolites

Antimetabolites such as azathioprine and mycophenolate mofetil (MMF) are widely used to prevent transplant rejection and treat autoimmune diseases (52). Azathioprine works by inhibiting purine metabolism, thus reducing DNA synthesis and suppressing the activity of immune cells like T-cells and B-cells. MMF, which blocks inosine monophosphate dehydrogenase, also impedes purine synthesis, but it tends to have fewer side effects than azathioprine (52). Both medications can cause bone marrow suppression, gastrointestinal distress, and an increased risk of infections and cancers. MMF, while generally preferred for its favourable safety profile (52), is contraindicated in pregnancy (FDA class D) due to teratogenicity (52). Antimetabolites (azathioprine, MMF) remain core agents in multimodal regimens (52).

#### 3.5.1 Biologic agents and monoclonal antibodies

Monoclonal antibodies and other biologic agents are essential components of immunosuppressive regimens, which are particularly reserved for induction therapy and the management of rejection reactions. These drugs work by targeting specific immune cells or receptors, such as interleukin-2 receptors and CD52, providing targeted immune modulation. The following section discusses different biological agents and their clinical roles in solid organ transplantation:

#### 3.5.2 Anti-thymocyte globulin (ATG)

Anti-thymocyte globulin (ATG) is a potent immunosuppressive therapy used to deplete T-cells in high-risk transplant recipients or those with acute rejection. Derived from animal serum (usually rabbit or horse), ATG binds to T-cell surface markers and induces their destruction through immune-mediated processes. This drug is particularly useful in preventing or treating acute graft rejection, but its use is associated with side effects such as fever, chills, and an increased risk of infections. ATG also increases the likelihood of serum sickness due to the immune response against the foreign antibodies used in the treatment (53).

#### 3.5.3 IL-2 receptor antagonists (e.g., basiliximab)

Basiliximab blocks IL-2 receptor activation, preventing T-cell activation rather than proliferation (54, 55).IL-2 is crucial for T-cell expansion and immune activation, and by inhibiting its receptor, basiliximab prevents the proliferation of T-cells, which helps to reduce the risk of transplant rejection (54). IL-2 receptor antagonists have the advantage of being less toxic compared to other immunosuppressive drugs like anti-thymocyte globulin. Side effects are generally mild, including infusion reactions like fever or chills. Basiliximab is often used in conjunction with other immunosuppressive therapies, especially in the early stages post-transplant (55).

#### 3.5.4 Co-Stimulation blockers

There are several co-stimulation blockers for immunosuppression. Examples include abatacept, FR104, and belatacept. However, the prominent blocker among them is Belatacept. Belatacept prevents T-cell activation by interfering with the CD28 pathway. It is contraindicated in EBV-negative patients due to the risk of post-transplant lymphoproliferative disorder and is not approved for *de novo* use in liver transplantation (56, 57). A significant advantage of belatacept over traditional calcineurin inhibitors is its reduced nephrotoxicity, which makes it ideal for long-term use. However, belatacept carries a higher risk of viral infections, particularly from Epstein–Barr virus (EBV), and is typically used in patients who are EBV-positive. It's often combined with other immunosuppressive agents to optimize transplant outcomes (57).

#### 3.5.5 Anti-CD20 agents

Various anti-CD20 agents, such as obinutuzumab, rituximab, and ofatumumab, are currently used to prevent antibody-mediated rejection. Of these, rituximab is the most commonly utilised anti-CD20 agent. Rituximab is a monoclonal antibody that targets the CD20 protein on B-cells, which are responsible for antibody production in the immune system (58). By depleting these B-cells, rituximab helps to reduce the risk of antibody-mediated rejection in organ transplant patients. This drug is particularly useful for patients with high levels of donor-specific antibodies, which can lead to transplant rejection (58). While rituximab is generally well tolerated, it can cause infusion-related reactions, including fever, chills, and hypotension. Long-term use also increases the risk of infections and certain cancers, necessitating careful monitoring of patients receiving this treatment (59).

### 3.6 Emerging strategies in transplant immunology

Recent advances in transplant immunology have led to innovative approaches aimed at reducing rejection while preserving immune competence. Tolerogenic dendritic cells (tDCs) are engineered to promote immune tolerance rather than activation, and clinical trials suggest they can reduce alloreactivity and prolong graft survival (5, 60). Regulatory T-cell (Treg) therapies represent another promising avenue; Tregs can be expanded *ex vivo* and reinfused to suppress rejection. The ONE Study Consortium demonstrated the safety and potential efficacy of adoptive Treg therapy, supporting tolerance induction without long-term pharmacological immunosuppression (35, 61).

Gene therapy is also being explored to modulate immune responses. Techniques such as CRISPR/Cas9 and viral vector-mediated transfer are under investigation to induce immunoregulatory gene expression or silence pro-rejection pathways in immune cells and graft tissues. Although still experimental, these approaches hold potential for personalized immunosuppression (62, 63).

Nanotechnology offers another frontier, enabling targeted and sustained delivery of immunosuppressants with reduced systemic toxicity. Preclinical studies show nanoparticles can achieve local immunoregulation by directing agents to lymphoid or graft tissues (64, 65). Finally, renewed interest in the mTOR pathway has spurred the development of next-generation inhibitors. Sirolimus has demonstrated efficacy in reducing rejection and preserving renal function (66), while newer mTOR inhibitors and dual-pathway agents are being investigated to improve selectivity and minimize metabolic complications (67).

### 3.7 Challenges in immunosuppressive therapy

Immunosuppressive therapy remains the cornerstone of preventing allograft rejection (68). However, its use is associated with significant adverse effects, including cardiovascular, neurological, hepatic, renal, endocrine, and metabolic toxicities, which contribute to reduced life expectancy compared to the general population (68). Immunosuppressed patients are also at increased risk of malignancies such as lymphoma and Kaposi sarcoma (68), as well as infections ranging from opportunistic (Pneumocystis, fungi, tuberculosis) to life-threatening (COVID-19, sepsis) and viral pathogens including Cytomegalovirus (CMV), Hepatitis B virus (HBV), Epstein–Barr Virus (EBV), BK polyomavirus, norovirus, and Hepatitis E virus (HEV) (49). Management of infection often requires careful reduction of immunosuppression to restore immune function while preserving graft integrity, though limited evidence means clinicians frequently rely on judgment rather than standardized guidelines (49).

Adverse effects also impair quality of life (70) and contribute to non-adherence, reported in 20%–70% of transplant patients (69). Non-adherence increases the risk of acute rejection, graft failure, and antibody-mediated rejection, with younger patients particularly vulnerable (70). Sociodemographic, treatment-related, and psychological factors further exacerbate noncompliance (69). Pharmacogenomics offers potential to tailor regimens and reduce toxicity (71), though it is rarely implemented in routine practice (71).

Special populations present additional challenges. Most immunosuppressants are safe during pregnancy and lactation, some require monitoring and some are strictly contraindicated (72). As addressed in the reviews, pediatric patients face challenges in development and quality of life (73, 74, 75). Whereas, senescence-related changes in pharmacokinetics and comorbidities pose challenges for geriatric patients (76). These multifaceted challenges underscore the complexity of balancing efficacy, toxicity, and long-term safety in immunosuppressive management.

## 4 Discussion

Building on these emerging strategies, the following discussion provides an organ-specific synthesis of immunobiology, rejection phenotypes, and monitoring approaches, while also identifying shared principles across transplant types.

### 4.1 Kidney transplantation

Kidney transplantation is currently the most extensively studied form of solid organ transplantation, largely due to higher frequency procedures globally and the large body of available long-term data. Kidney transplant rejection is mainly mediated through both T-cell–mediated rejection (TCMR) and AMR. In TCMR, activated recipient T lymphocytes identify donor human leukocyte antigen (HLA) molecules through direct and indirect antigen presentation pathways. These activated T cells produce pro-inflammatory cytokines predominantly IL-2, IFN-*γ*, TNF-*α*, and IL-17, which in turn results in destruction of both renal tubular epithelial cells and the graft vasculature.

Histologically, TCMR is characterized by interstitial leukocyte infiltration, inflammation of renal tubules, and arteritis (77). In AMR, donor-specific antibodies (DSAs) are formed against the graft, which target donor HLA molecules expressed on endothelial cells. This results in the complement cascade activation and subsequent microvascular inflammation (78). AMR is characterized by C4d deposition within peritubular capillaries and endothelial injury, which can ultimately lead to chronic allograft nephropathy and graft loss (79). In recent years, non-invasive biomarkers have transformed graft monitoring in kidney transplantation. Donor-derived cell-free DNA (dd-cfDNA) has emerged as a novel, safe, and promising biomarker for detecting graft injury and early rejection. Elevated dd-cfDNA levels represent allograft damage and have high sensitivity for detecting active or ongoing rejection episodes (80). This strategy can complement traditional surveillance strategies such as serum creatinine measurement, donor-specific antibody testing, and allograft biopsy.

The Kidney Disease: Improving Global Outcomes (KDIGO) guidelines emphasize the precise management of kidney transplantation. Multiple factors, including HLA mismatch, recipient sensitization status, prior transplantation, and the presence of DSAs, are risk factors for rejection. Additionally, the selection and intensity of immunosuppressive therapy can be modified in the presence of recipient comorbidities such as diabetes, cardiovascular disease, and infection (81).

### 4.2 Liver transplantation

The liver occupies a unique position among solid organ transplants due to its relative immunologic tolerance compared with other graft types. Several factors make the liver relatively tolerant. First, the liver's rich population of antigen-presenting cells, such as Kupffer cells, liver sinusoidal endothelial cells, and regulatory dendritic cells. These cells regulate T-cell anergy, deletion, or the expansion of regulatory T cells (Tregs). Second, the liver's dual blood supply (hepatic artery and portal vein) and continuous exposure to gut-derived antigens contribute to the immunomodulatory environment that favors tolerance. As a result, the incidence of chronic rejection following liver transplantation is significantly lower than that observed in kidney, heart, or lung transplantation (82).

Liver transplant rejection is mainly driven by acute cellular rejection (ACR) and AMR. ACR remains the major cause of liver transplant rejection. It occurs particularly within the first weeks to months following transplantation in approximately 15%–30% of liver transplant recipients, and is highly variable based on recipient risk factors. Recipient T lymphocytes recognize donor HLA molecules through direct and indirect allorecognition pathways, which result in the release of pro-inflammatory cytokines, causing hepatic damage (83). Histologically, ACR is characterized by portal inflammation, bile duct injury, and endothelial inflammation within portal and central veins (84). In contrast, AMR is relatively rare in liver transplantation. The liver is relatively resistant to AMR, which may be due to its capacity to absorb circulating DSAs and the presence of complement regulatory proteins expressed on hepatic endothelial cells (85).

Diagnosis and monitoring of rejection in liver transplantation is facilitated via a combination of clinical assessment, laboratory testing, and histological evaluation. Elevation in serum liver enzymes is the most common early indicator of graft injury, but it is usually nonspecific and may reflect other liver diseases. Therefore, liver biopsy is the gold standard for detecting liver transplant rejection (83). Non-invasive investigational biomarkers include dd-cfDNA, gene expression profiling, circulating microRNAs, and immune cell signatures, which may provide earlier and more specific detection of rejection risk (86). Recent American Society of Transplantation (AST) and American Association for the Study of Liver Diseases (AASLD) guidelines highlight candidate evaluation and graft-related complications, highlighting the importance of individualized monitoring strategies in liver recipients (87).

### 4.3 Heart transplantation

Heart transplantation is the definitive therapy for patients with advanced heart failure refractory to medical and device-based therapies. However, the long-term graft and patient survival is mainly limited by AMR and cardiac allograft vasculopathy (CAV) (88). The International Society for Heart and Lung Transplantation (ISHLT) recommends regular post-transplant surveillance for DSAs, as both pre-existing and *de novo* DSAs are strongly associated with AMR, CAV development, and late graft dysfunction. Monitoring for anti-HLA antibodies is typically performed at 1, 3, 6, and 12 months after transplantation and periodically thereafter for early detection of immunologic sensitization (89). Histologically, AMR is characterized by myocardial capillary C4d deposition, accompanied by macrophage accumulation and interstitial edema (90).

Meanwhile, CAV remains the leading cause of late graft failure after heart transplantation. CAV is a diffuse and progressive form of transplant vasculopathy that is characterized by concentric intimal proliferation in epicardial and intramyocardial arteries. Both AMR and TCMR contribute to CAV progression (91).

Endomyocardial biopsy (EMB) has been the gold standard traditional monitoring method. However, its use remains limited owing to significant interobserver variability in the pathologic diagnosis and invasive nature (92). Non-invasive biomarkers for heart transplantation are mainly dd-cf DNA and DSAs. However, DSAs alone do not consistently predict rejection, as a substantial proportion of DSA-positive patients do not develop histologic AMR (93). Moreover, studies show significantly reduced survival in patients with both pathological AMR and circulating DSAs compared with DSA-negative recipients (94), which highlights the crucial need for integrated diagnostic frameworks combining immunologic, molecular, and functional markers.

Current management strategies to prevent heart transplantation rejection mainly comprise nonspecific immunosuppression (calcineurin inhibitors, antimetabolites, and corticosteroids), while adjunctive therapies (such as plasmapheresis, intravenous immunoglobulin, rituximab, and proteasome inhibitors) are reserved for refractory AMR (95). Emerging strategies aim towards targeted immune modulation, such as costimulation blockade (e.g., agents targeting CD28–CD80/86 signaling), which is under investigation (96). Despite these advances, there remains the need for therapies capable of preventing both acute AMR and CAV without excessive immunosuppression, which should be the focus of future studies.

### 4.4 Lung transplantation

Chronic lung allograft dysfunction (CLAD) remains the leading cause of late graft failure and mortality among lung transplant recipients. CLAD refers to progressive and irreversible decline in graft function, most commonly categorized into bronchiolitis obliterans syndrome (BOS) and restrictive allograft syndrome (RAS) (97). CLAD represents a complex interaction between immune-mediated rejection and chronic inflammatory injury. Both ACR and AMR induce repeated episodes of epithelial and endothelial injury, which results in chronic fibroproliferative remodeling and subsequent CLAD development (98). Non-alloimmune factors, including primary graft dysfunction, respiratory infections, gastroesophageal reflux, and environmental exposures, have been implicated in CLAD pathogenesis. These factors promote persistent airway inflammation and epithelial injury, which in turn trigger CLAD development (99).

Post-transplant monitoring involves routine surveillance, which includes serial spirometry (100). Bronchoscopy with bronchoalveolar lavage and transbronchial biopsy is frequently employed to evaluate suspected rejection or infection, although its use is limited due to the invasive nature of the procedure (101). Radiographic assessment with high-resolution computed tomography can also help differentiate obstructive and restrictive CLAD phenotypes, particularly in the evaluation of RAS (102). The emerging molecular biomarkers include dd-cfDNA assays with elevated levels correlating with acute rejection, infection, and early CLAD development. However, its role in routine surveillance protocols continues to be defined due to its high negative predictive value (103).

**Figure 4:**
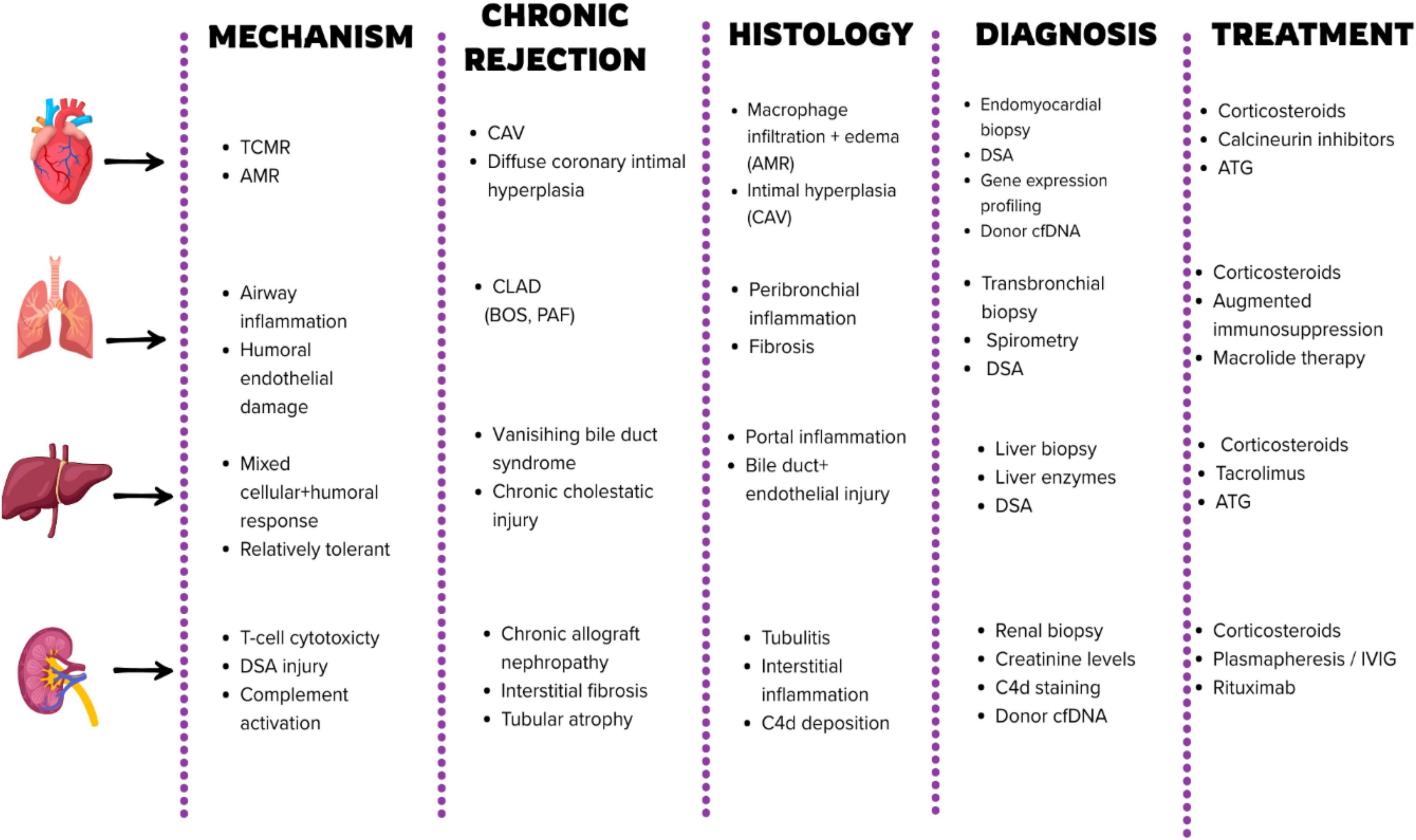
Mechanisms, chronic rejection patterns, histology, diagnosis, and treatment across major solid organ transplants (tCMR, T-cell mediated rejection; AMR, antibody-mediated rejection; CAV, cardiac allograft vasculopathy; cLAD, chronic lung allograft dysfunction; BOS, bronchiolitis obliterans syndrome; PAF, pulmonary allograft fibrosis; DSA, donor-specific antibodies; cfDNA, cell-free DNA; ATG, anti-thymocyte globulin; C4d, C4d deposition.

Despite recent advances, long—term outcomes after lung transplantation remain inferior compared with other solid organ transplants (104). Current consensus statements from the International Society for Heart and Lung Transplantation (ISHLT) emphasize early rejection detection and understanding chronic graft injury, integrating biomarkers with monitoring, and developing targeted therapies to prevent CLAD (105) (Figure 4).

### 4.5 Vascularized composite allografts (Vca)

Vascularized composite allotransplantation (VCA) poses unique immunologic and ethical challenges. It involves the transplantation of multiple tissue types (such as skin, muscle, bone, nerves, and blood vessels) as a functional unit. It may be taken from the face, hand, uterus, and genitalia and is mainly aimed at improving aesthetics and function (106). Recipients face a particularly high risk of AMR, yet current monitoring relies heavily on invasive biopsies that may miss subclinical injury. Because VCA is generally non-life-saving, it raises significant concerns regarding the risk–benefit balance, given the need for lifelong immunosuppression and associated risk of infection, malignancy, and metabolic complications (107). Novel options such as cffDNA and immunologic profiling show potential but remain inconsistently predictive. Strategies such as tolerance induction and gene therapy could reduce the immunosuppressive burden; however, these approaches are not standardized in clinical practice. The lack of standardized guidelines underscores uncertainty in management, with long-term outcomes largely dependent on center expertise and individualized treatment.

### 4.6 Integrative perspective

While organ-specific differences are critical, shared principles such as the role of alloimmune responses, the utility of cfDNA, and the promise of tolerance-inducing therapies underscore the value of a cross-organ synthesis. This comparative lens complements organ-specific reviews by offering clinicians a unified framework that highlights both commonalities and unique challenges across kidney, liver, heart, lung, and VCA transplantation.

Despite these advances, several challenges remain in the management of graft rejection. The heterogeneity of rejection mechanisms across different organ types and patient populations complicates the development of universally effective treatment strategies. Additionally, long-term immunosuppression continues to carry significant health risks, emphasizing the need for safer and more targeted therapies. Future research should focus on developing novel immunomodulatory strategies that promote immune tolerance while minimizing systemic immunosuppression.

## 5 Novel preventive and therapeutic approaches

Although the pathological phenotypes and mechanisms of graft rejection remain incompletely understood, several innovative strategies are being explored to reduce their incidence. Clinical trials suggest that the proteasome inhibitor bortezomib, in combination with plasmapheresis, can induce apoptosis and lower anti-HLA antibody levels in renal graft rejection (64). Personalized agents such as atacicept and belimumab, which target B-cell survival and amplification, are also in clinical development (65). These approaches highlight the need for continued clinical trials focused on personalized interventions to address persistent challenges in rejection management.

Future strategies emphasize AI-driven diagnostics, biomarker-guided immunosuppression, and immune tolerance induction (59–62). Emerging fields include vascularized composite allografts (face, hand, genitalia) and xenotransplantation, where antibody barriers such as *α*-gal remain critical obstacles (65–67). Gene therapy is likewise under investigation as a tool to modulate immune responses (68). The development of non-invasive biomarkers represents another breakthrough, with CD68 + and CD163 + macrophages identified as prognostic markers in chronic rejection (108, 109).

Tolerance-based therapies remain a central goal. Antigen-specific approaches using Tregs, mesenchymal stem cells, and regulatory dendritic cells have shown promise in preventing rejection (110). Nanoparticles offer a novel means of directing immune responses by targeting antigen-presenting cells (111), and early reports on Treg therapy suggest encouraging safety profiles in graft recipients (50). Advances in biotechnology and tissue engineering have also enabled the development of bioengineered grafts designed to reduce immune activation and improve survival. Machine learning algorithms are increasingly applied to graft allocation, survival prediction, and postoperative risk assessment (112), while cell-based therapies and AI-driven decision support hold promise for optimizing patient outcomes (113). Collectively, these innovations underscore the need for ongoing research to improve long-term graft survival and patient health.

## 6 Study limitations

This review has several limitations. First, although we rebuilt the methodology to align with PRISMA standards, the heterogeneity of available studies, ranging from randomized trials to observational cohorts and preclinical reports, limited the ability to perform meta-analysis. Second, the evidence base is uneven across organs: kidney transplantation is extensively studied, whereas liver, heart, lung, and VCA have fewer high-quality data, which may skew emphasis. Third, despite formal risk-of-bias assessment, many included studies had small sample sizes or short follow-up, reducing certainty in long-term outcomes. Finally, transplantation research is rapidly evolving; advances such as donor-derived cfDNA monitoring, machine perfusion, and AI-driven diagnostics continue to emerge, and our synthesis captures evidence only up to June 2025. These limitations underscore the need for ongoing systematic updates and organ-specific trials to strengthen the evidence base.

## 7 Conclusion

Graft rejection remains a central challenge in transplant medicine, driven by complex interactions among T cells, B cells, alloantigen recognition, and inflammatory pathways. Advances in immunosuppressive therapy from corticosteroids to biologics and cellular approaches have improved survival, yet adverse drug effects, infection risk, and patient adherence continue to limit outcomes. While strategies such as gene therapy, biomarker-guided monitoring, and nanotechnology show promise, many have already been extensively reviewed in recent consensus statements.

The distinctive contribution of this study lies in its organ-specific synthesis, highlighting differences in immunobiology, rejection phenotypes, and monitoring strategies across kidney, liver, heart, lung, and vascularized composite allografts. Future progress will depend on refining tolerance-inducing approaches, integrating evidence-based biomarkers, and advancing bioengineered grafts. Continued research, guided by consensus frameworks, is essential to translate these innovations into safer and more durable transplant outcomes.

## Data Availability

The original contributions presented in the study are included in the article/Supplementary Material, further inquiries can be directed to the corresponding author.

## References

[1] Kidney Disease: Improving Global Outcomes (KDIGO) Transplant Work Group. Kdigo clinical practice guideline for the care of kidney transplant recipients. Am J Transplant. (2009) 9(Suppl 3):S1–155. 10.1111/j.1600-6143.2009.02834.x 19845597

[2] Taylor AL Watson CJE Bradley JA. Immunosuppressive agents in solid organ transplantation: mechanisms of action and therapeutic efficacy. Crit Rev Oncol Hematol. (2005) 56(1 SPEC. ISS.):23–46. 10.1016/j.critrevonc.2005.03.012 16039869

[3] Van Kooten C Lombardi G Gelderman KA. Dendritic cells as a tool to induce transplantation tolerance: obstacles and opportunities. Transplantation. (2011) 91(1):2–7. 10.1097/TP.0B013E31820263B3 21452405

[4] Clayton PA McDonald SP Russ GR Chadban SJ. Long-term outcomes after acute rejection in kidney transplant recipients: an ANZDATA analysis. J Am Soc Nephrol. (2019) 30(9):1697–1707. 10.1681/ASN.2018111101 31308074 PMC6727270

[5] Ochando J Ordikhani F Jordan S Boros P Thomson AW. Tolerogenic dendritic cells in organ transplantation. Transplant Int. (2020) 33(2):113–127. 10.1111/tri.13504 PMC6983332 31472079

[6] Abu-Elmagd KM Costa G Bond GJ. Evolution of the immunosuppressive strategies for the intestinal and multivisceral recipients with special reference to allograft immunity and achievement of partial tolerance. Transpl Int. (2009) 22(1):96–109. 10.1111/J.1432-2277.2008.00785.X 18954362

[7] Pilch NA Bowman LJ Taber DJ. Immunosuppression trends in solid organ transplantation: the future of individualization, monitoring, and management. Pharmacotherapy. (2021) 41(1):119–131. 10.1002/PHAR.2481 33131123 PMC8778961

[8] Anwar IJ Berman DM DeLaura I. The anti-CD40L monoclonal antibody AT-1501 promotes islet and kidney allograft survival and function in nonhuman primates. Sci Transl Med. (2023) 15(711). 10.1126/SCITRANSLMED.ADF6376 37647390 PMC10990482

[9] Yakubu I Moinuddin I Brown A Sterling S Sinhmar P Kumar D. Costimulation blockade: the next generation. Curr Opin Organ Transplant. (2025) 30(2). 10.1097/MOT.0000000000001206 39882641

[10] Vaillant AAJ Mohseni M. Chronic Transplantation Rejection. Treasure Island, Florida: StatPearls (2024). Available online at: https://www.ncbi.nlm.nih.gov/books/NBK535435/ (Accessed June 10, 2025).

[11] Harmon WE. Pediatric Renal Transplantation. Chronic Kidney Disease, Dialysis, and Transplantation: A Companion to Brenner and Rector’s the Kidney - Expert Consult. Philadelphia, Pennsylvania: Elsevier (2010). p. 591–608. 10.1016/B978-1-4377-0987-2.00041-8

[12] Chih S Chruscinski A Ross HJ Tinckam K Butany J Rao V. Antibody-mediated rejection: an evolving entity in heart transplantation. J Transplant. (2012) 2012:210210. 10.1155/2012/210210 22545200 PMC3321610

[13] Naik RH Shawar SH. Renal Transplantation Rejection. Treasure Island, Florida: StatPearls Publishing (2023). p. 1. Available online at: https://www.ncbi.nlm.nih.gov/books/NBK553074/ (Accessed June 10, 2025).

[14] Becker JU Seron D Rabant M Roufosse C Naesens M. Evolution of the definition of rejection in kidney transplantation and its use as an endpoint in clinical trials. Transpl Int. (2022) 35:10141. 10.3389/TI.2022.10141 35669978 PMC9163319

[15] Carnel N Lancia HH Guinier C Benichou G. Pathways of antigen recognition by T cells in allograft rejection. Transplantation. (2022) 107(4):827. 10.1097/TP.0000000000004420 36398330 PMC10600686

[16] Li X Zhuang S. Recent advances in renal interstitial fibrosis and tubular atrophy after kidney transplantation. Fibrogenesis Tissue Repair. (2014) 7(1):15. 10.1186/1755-1536-7-15 25285155 PMC4185272

[17] Krishna R Anjum F Oliver TI. Bronchiolitis Obliterans. Treasure Island, Florida: StatPearls (2023). Available online at: https://www.ncbi.nlm.nih.gov/books/NBK441865/ (Accessed June 10, 2025).

[18] Hubscher SG Buckels JAC Elias E McMaster P Neuberger J. Vanishing bile-duct syndrome following liver transplantation-is it reversible? Transplantation. (1991) 51(5):1004–1010. 10.1097/00007890-199105000-00014 1851580

[19] Pober JS Chih S Kobashigawa J Madsen JC Tellides G. Cardiac allograft vasculopathy: current review and future research directions. Cardiovasc Res. (2021) 117(13):2624–2638. 10.1093/CVR/CVAB259 34343276 PMC8783389

[20] Kalaria AL Yamada T Klein-Fedyshin M. Subclinical rejection and allograft survival in kidney transplantation: protocol for a systematic review and meta-analysis. BMJ Open. (2024) 14(7):e085098. 10.1136/BMJOPEN-2024-085098 39025816 PMC11261677

[21] Salvadori M Rosso G Bertoni E. Update on ischemia-reperfusion injury in kidney transplantation: pathogenesis and treatment. World J Transplant. (2015) 5(2):52. 10.5500/WJT.V5.I2.52 26131407 PMC4478600

[22] Hassanein M Augustine JJ. Chronic Kidney Transplant Rejection. Treasure Island, Florida: StatPearls (2023). Available online at: https://www.ncbi.nlm.nih.gov/books/NBK549762/ (Accessed June 10, 2025). 31747169

[23] Pittappilly M Sharshir M Paramesh A. Chronic allograft nephropathy—a narrative review of its pathogenesis, diagnosis, and evolving management strategies. Biomedicines. (2025) 13(4):929. 10.3390/BIOMEDICINES13040929 40299546 PMC12024747

[24] Ingulli E. Mechanism of cellular rejection in transplantation. Pediatr Nephrol. (2010) 25(1):61. 10.1007/S00467-008-1020-X 21476231 PMC2778785

[25] Akatsuka Y Nishida T Kondo E. Identification of a polymorphic gene, BCL2A1, encoding two novel hematopoietic lineage-specific Minor histocompatibility antigens. J Exp Med. (2003) 197(11):1489. 10.1084/JEM.20021925 12771180 PMC2193899

[26] Simpson E Scott D Chandler P. The male-specific histocompatibility antigen, H-Y: a history of transplantation, immune response genes, sex determination and expression cloning. Annu Rev Immunol. (1997) 15:39–61. 10.1146/ANNUREV.IMMUNOL.15.1.39 9143681

[27] Spierings E Vermeulen CJ Vogt MH. Identification of HLA class II-restricted H-Y-specific T-helper epitope evoking CD4+ T-helper cells in H-Y-mismatched transplantation. Lancet. (2003) 362(9384):610–615. 10.1016/S0140-6736(03)14191-8 12944060

[28] Roopenian D Young Choi E Brown A. The immunogenomics of minor histocompatibility antigens. Immunol Rev. (2002) 190:86–94. 10.1034/J.1600-065X.2002.19007.X 12493008

[29] Haskova Z Sproule TJ Roopenian DC Ksander BR. An immunodominant minor histocompatibility alloantigen that initiates corneal allograft rejection. Transplantation. (2003) 75(8):1368–1374. 10.1097/01.TP.0000063708.26443.3B 12717232

[30] Gago M Cornell LD Kremers WK Stegall MD Cosio FG. Kidney allograft inflammation and fibrosis: causes and consequences. Am J Transplant. (2012) 12(5):1199–1207. 10.1111/J.1600-6143.2011.03911.X 22221836

[31] Karahan GE Claas FHJ Heidt S. B-cell immunity in solid organ transplantation. Front Immunol. (2017) 7. 10.3389/FIMMU.2016.00686 28119695 PMC5222792

[32] Huang C Gonzalez DG Cote CM. The BCL6 RD2 domain governs commitment of activated B cells to form germinal centers. Cell Rep. (2014) 8(5):1497–1508. 10.1016/J.CELREP.2014.07.059 25176650 PMC4163070

[33] Jindra PT Jin YP Rozengurt E Reed EF. Hla class I antibody-mediated endothelial cell proliferation via the mTOR pathway. J Immunol. (2008) 180(4):2357–2366. 10.4049/JIMMUNOL.180.4.2357 18250445

[34] Valenzuela NM McNamara JT Reed EF. Antibody-mediated graft injury: complement-dependent and complement-independent mechanisms. Curr Opin Organ Transplant. (2014) 19(1):33–40. 10.1097/MOT.0000000000000040 24316758 PMC4080796

[35] Sawitzki B Harden PN Reinke P. Regulatory cell therapy in kidney transplantation (the ONE study). Lancet. (2020) 395(10237):1627–1639. 10.1016/S0140-6736(20)30167-7 32446407 PMC7613154

[36] Philogene MC Jackson AM. Non-HLA antibodies in transplantation: when do they matter? Curr Opin Organ Transplant. (2016) 21(4):427–432. 10.1097/MOT.0000000000000335 27258575

[37] Pouliquen E Koenig A Chen CC. Recent advances in renal transplantation: antibody-mediated rejection takes center stage. F1000Prime Rep. (2015) 7:51. 10.12703/P7-51 26097724 PMC4447042

[38] Hafeez MS Awais SB Razvi M. HLA mismatch is important for 20-year graft survival in kidney transplant patients. Transpl Immunol. (2023):80. 10.1016/j.trim.2023.101861 37302557

[39] Hosenpud JD Edwards EB Lin HM Daily OP. Influence of HLA matching on thoracic transplant outcomes: an analysis from the UNOS/ISHLT thoracic registry. Circulation. (1996) 94(2):170–174. 10.1161/01.CIR.94.2.170 8674175

[40] Osorio-Jaramillo E Haasnoot GW Kaider A. Molecular-level HLA mismatch is associated with rejection and worsened graft survival in heart transplant recipients – a retrospective study. Transpl Int. (2020) 33(9):1078–1088. 10.1111/TRI.13657 32441827 PMC7540475

[41] Toyoda M Pao A Vo A. Intracellular IFN*γ* production in CD3-negative cells exposed to allo-antigens is an indicator of prior sensitization. Transpl Immunol. (2010) 22(3-4):121–127. 10.1016/j.trim.2009.11.004 19944759

[42] Glotz D Antoine C Julia P. Intravenous immunoglobulins and transplantation for patients with anti-HLA antibodies. Transpl Int. (2004) 17(1):1–8. 10.1111/J.1432-2277.2004.TB00376.X/PDF 14685653

[43] Lund LH Khush KK Cherikh WS. The registry of the international society for heart and lung transplantation: thirty-fourth adult heart transplantation report—2017; focus theme: allograft ischemic time. J Heart Lung Transplant. (2017) 36(10):1037–1046. 10.1016/J.HEALUN.2017.07.019 28779893

[44] Nair N. Vascular rejection in cardiac allograft vasculopathy: impact on graft survival. Front Cardiovasc Med. (2022):9. 10.3389/FCVM.2022.919036 PMC9386065 35990962

[45] Koo DDH Welsh KI Roake JA Morris PJ Fuggle SV. Ischemia/reperfusion injury in human kidney transplantation: an immunohistochemical analysis of changes after reperfusion. Am J Pathol. (1998) 153(2):557–566. 10.1016/S0002-9440(10)65598-8 9708815 PMC1852972

[46] Barba J Zudaire JJ Robles JE. ¿Existe un intervalo seguro de isquemia fría para el injerto renal? Actas Urol Esp. (2011) 35(8):475–480. 10.1016/J.ACURO.2011.03.005 21550140

[47] Eide IA Halden TAS Hartmann A Dahle DO Åsberg A Jenssen T. Associations between posttransplantation diabetes mellitus and renal graft survival. Transplantation. (2017) 101(6):1282–1289. 10.1097/TP.0000000000001259 27362306

[48] Wojciechowski D Wiseman A. Long-Term immunosuppression management: opportunities and uncertainties. Clin J Am Soc Nephrol. (2021) 16(8):1264. 10.2215/CJN.15040920 33853841 PMC8455033

[49] Roberts MB Fishman JA. Immunosuppressive agents and infectious risk in transplantation: managing the “net state of immunosuppression”. Clin Infect Dis. (2021) 73(7):E1302–E1317. 10.1093/CID/CIAA1189 32803228 PMC8561260

[50] Pilat N Steiner R Sprent J. Treg therapy for the induction of immune tolerance in transplantation—not lost in translation? Int J Mol Sci. (2023) 24(2):21752. 10.3390/ijms24021752 PMC9861925 36675265

[51] Naesens M Kuypers DRJ Sarwal M. Calcineurin inhibitor nephrotoxicity. Clin J Am Soc Nephrol. (2009) 4(2):481–508. 10.2215/CJN.04800908 19218475

[52] Srinivas TR Kaplan B Meier-Kriesche HU. Mycophenolate mofetil in solid-organ transplantation. Expert Opin Pharmacother. (2003) 4(12):2325–2345. 10.1517/14656566.4.12.2325 14640931

[53] Acharya S Lama S Kanigicherla DA. Anti-thymocyte globulin for treatment of T-cell-mediated allograft rejection. World J Transplant. (2023) 13(6):299–308. 10.5500/WJT.V13.I6.299 38174145 PMC10758678

[54] Sageshima J Ciancio G Chen L Burke GW 3rd. Anti-interleukin-2 receptor antibodies-basiliximab and daclizumab-for the prevention of acute rejection in renal transplantation. Biologics. (2009) 3:319–36. 10.2147/btt.2009.3257 19707418 PMC2726067

[55] Bae S Durand CM Garonzik-Wang JM. Antithymocyte globulin versus interleukin-2 receptor antagonist in kidney transplant recipients with hepatitis C virus. Transplantation. (2020) 104(6):1294–1303. 10.1097/TP.0000000000002959 32433232 PMC7534413

[56] Parsons RF Larsen CP Pearson TC Badell IR. Belatacept and CD28 costimulation blockade: preventing and reducing alloantibodies over the long term. Curr Transplant Rep. (2019) 6(4):277. 10.1007/S40472-019-00260-3 32158639 PMC7063534

[57] Kumar D LeCorchick S Gupta G. Belatacept as an alternative to calcineurin inhibitors in patients with solid organ transplants. Front Med (Lausanne). (2017) 4:60. 10.3389/FMED.2017.00060 28580358 PMC5437176

[58] Selewski DT Shah GV Mody RJ Rajdev PA Mukherji SK. Rituximab (rituxan). Am J Neuroradiol. (2010) 31(7):1178–1180. 10.3174/AJNR.A2142 20448016 PMC7965451

[59] Ghrenassia E Mariotte E Azoulay E. Rituximab-related severe toxicity. In: Vincent JL, editor. Annual Update in Intensive Care and Emergency Medicine. Cham, Switzerland: Springer International Publishing (Springer Nature) (2018). p. 579. 10.1007/978-3-319-73670-9_43

[60] Li H Shi B. Tolerogenic dendritic cells and their applications in transplantation. Cell Mol Immunol. (2015) 12(1):24–30. 10.1038/CMI.2014.52 25109681 PMC4654373

[61] Guinan EC Contreras-Ruiz L Crisalli K. Donor antigen-specific regulatory T cell administration to recipients of live donor kidneys: a ONE study consortium pilot trial. Am J Transplant. (2023) 23(12):1872–1881. 10.1016/j.ajt.2023.06.012 37422112

[62] Lisjak M De Caneva A Marais T. Promoterless gene targeting approach combined with CRISPR/Cas9 efficiently corrects haemophilia B phenotype in neonatal mice. Front Genome Ed. (2022) 4. 10.3389/FGEED.2022.785698 35359664 PMC8962648

[63] Taha EA Lee J Hotta A. Delivery of CRISPR-cas tools for *in vivo* genome editing therapy: trends and challenges. J Controlled Release. (2022) 342:345–361. 10.1016/J.JCONREL.2022.01.013 35026352

[64] Lukin I Erezuma I Desimone MF Zhang YS Dolatshahi-Pirouz A Orive G. Nanomaterial-based drug delivery of immunomodulatory factors for bone and cartilage tissue engineering. Biomaterials Advances. (2023) 154:213637. 10.1016/J.BIOADV.2023.213637 37778293

[65] Jahanban-Esfahlan R Seidi K Zarghami N. Tumour vascular infarction: prospects and challenges. Int J Hematol. (2017) 105(3):244–256. 10.1007/S12185-016-2171-3 28044258

[66] Gaber AO Kahan BD Van Buren C Schulman SL Scarola J Neylan JF. Comparison of sirolimus plus tacrolimus versus sirolimus plus cyclosporine in high-risk renal allograft recipients: results from an open-label, randomized trial. Transplantation. (2008) 86(9):1187–1195. 10.1097/TP.0B013E318187BAB0 19005398

[67] Ali ES Mitra K Akter S. Recent advances and limitations of mTOR inhibitors in the treatment of cancer. Cancer Cell Int. (2022) 22(1). 10.1186/S12935-022-02706-8 PMC9476305 36109789

[68] Claeys E Vermeire K Leuven KU. Immunosuppressive drugs in organ transplantation to prevent allograft rejection: mode of action and side effects. J Immunol Sci. (2019) 3(4):14–21. 10.29245/2578-3009/2019/4.1178

[69] Wagner-Skacel J Fink N Kahn J. Improving adherence to immunosuppression after liver or kidney transplantation in individuals with impairments in personality functioning – A randomized controlled single-center feasibility study. Front Psychol. (2023) 14:1150548. 10.3389/FPSYG.2023.1150548/BIBTEX 36968754 PMC10033957

[70] Bethany JF Ahna LHP. Adherence in adolescent and young adult kidney transplant recipients. Open Urol Nephrol J. (2019) 7(1):133–143. 10.2174/1874303X014070100133

[71] Turolo SE defonti A Syren ML Montini G. Pharmacogenomics of old and new immunosuppressive drugs for precision medicine in kidney transplantation. J Clin Med. (2023) 12(13):4454. 10.3390/JCM12134454 37445489 PMC10342352

[72] Saad AF Pacheco LD Saade GR. Immunosuppressant Medications in Pregnancy. Obstet Gynecol. (2024) 143(4):e94–e106. 10.1097/AOG.0000000000005512 38227938

[73] Schonder KS Mazariegos GV Weber RJ. Adverse effects of immunosuppression in pediatric solid organ transplantation. Paediatr Drugs. (2010) 12(1):35–49. 10.2165/11316180-000000000-00000 20034340

[74] Zicarelli M Errante A Andreucci M Coppolino G Bolignano D. Immunosuppressive Therapy, Puberty and Growth Outcomes in Pediatric Kidney Transplant Recipients: A Pragmatic Review. Pediatr Transplant. (2024) 28(7):e14878. 10.1111/petr.14878 39445392

[75] Miloh T Barton A Wheeler J. Immunosuppression in pediatric liver transplant recipients: Unique aspects. Liver Transpl. (2017) 23(2):244–256. 10.1002/lt.24677 27874250

[76] Krenzien F El Hajj S Tullius SG Gabardi S. (2019). Immunosenescence and immunosuppressive drugs in the dlderly. Handbook of Immunosenescence. 2147–67. 10.1007/978-3-319-99375-1_137

[77] Lusco MA Fogo AB Najafian B Alpers CE. Ajkd atlas of renal pathology: acute T-cell–mediated rejection. Am J Kidney Dis. (2016) 67(5):e29–30. 27091022 10.1053/j.ajkd.2016.03.004

[78] Rodriguez-Ramirez S Al Jurdi A Konvalinka A Riella LV. Antibody-mediated rejection: prevention, monitoring and treatment dilemmas. Curr Opin Organ Transplant. (2022) 27(5):405–14. 10.1097/MOT.0000000000001011 35950887 PMC9475491

[79] Haas M Loupy A Lefaucheur C Roufosse C Glotz D Seron D. The banff 2017 kidney meeting report: revised diagnostic criteria for chronic active T cell–mediated rejection, antibody-mediated rejection, and prospects for integrative endpoints for next-generation clinical trials. Am J Transplant. (2018) 18(2):293–307. 10.1111/ajt.14625 29243394 PMC5817248

[80] Benning L Morath C Fink A Rudek M Speer C Kälble F. Donor-derived cell-free DNA (dd-cfDNA) in kidney transplant recipients with indication biopsy-results of a prospective single-center trial. Transpl Int. (2023) 36:11899. 10.3389/ti.2023.11899 38020751 PMC10654198

[81] Stevens PE Ahmed SB Carrero JJ Foster B Francis A Hall RK. Kdigo 2024 clinical practice guideline for the evaluation and management of chronic kidney disease. Kidney Int. (2024) 105(4):S117–314. 10.1016/j.kint.2023.10.018 38490803

[82] Zheng M Tian Z. Liver-mediated adaptive immune tolerance. Front Immunol. (2019):10. 10.3389/fimmu.2019.02525 31787967 PMC6856635

[83] Choudhary NS Saigal S Bansal RK Saraf N Gautam D Soin AS. Acute and chronic rejection after liver transplantation: what A clinician needs to know. J Clin Exp Hepatol. (2017) 7(4):358–66. Available online at: https://www.sciencedirect.com/science/article/pii/S097368831730467X (Accessed June 10, 2025). 29234201 10.1016/j.jceh.2017.10.003 PMC5715482

[84] Vasuri F Malvi D D’Errico A. Liver. Abdominal Solid Organ Transplantation. Cham, Switzerland: Springer Nature (2015). p. 185–207.

[85] Kozlowski T Rubinas T Nickeleit V Woosley J Schmitz J Collins D. Liver allograft antibody-mediated rejection with demonstration of sinusoidal C4d staining and circulating donor-specific antibodies. Liver Transpl. (2011) 17(4):357–68. 10.1002/lt.22233 21445918

[86] Julian J Millán O Titos E Ruiz P Fundora Y Díaz A. Donor-derived cell-free DNA and miRNA monitoring for the early prediction and diagnosis of liver allograft rejection and patient outcomes. Front Immunol. (2025) 16:1604200. 10.3389/fimmu.2025.1604200 40630960 PMC12234302

[87] Verna E. Highlights from the recent AASLD AST practice guideline on adult liver transplantation and graft-related complications. Gastroenterol Hepatol (N Y). (2025) 21(12):773. Available online at: https://pmc.ncbi.nlm.nih.gov/articles/PMC12872272 (Accessed June 10, 2025). 41647470 PMC12872272

[88] Mehta A DeFilippis EM Stehlik J Jackson AM Kobashigawa JA Shah P. Contemporary antibody-mediated rejection in heart transplantation: JACC: heart failure position statement. JACC Heart Fail. (2025) 13(10):102614. 10.1016/j.jchf.2025.102614 40848703 PMC13537435

[89] Kobashigawa J Colvin M Potena L Dragun D Crespo-Leiro MG Delgado JF. The management of antibodies in heart transplantation: an ISHLT consensus document. J Heart Lung Transplant. (2018) 37(5):537–47. 10.1016/j.healun.2018.01.1291 29452978

[90] Fedrigo M Leone O Burke MM Rice A Toquet C Vernerey D. Inflammatory cell burden and phenotype in endomyocardial biopsies with antibody-mediated rejection (AMR): a multicenter pilot study from the AECVP. Am J Transplant. (2015) 15(2):526–34. 10.1111/ajt.12976. 25612500

[91] Stomberski CT Colvin MM. Cardiac allograft vasculopathy: a focus on advances in diagnosis and management. Methodist Debakey Cardiovasc J. (2025) 21(3):58–71. 10.14797/mdcvj.1580 40384732 PMC12082475

[92] Bermpeis K Esposito G Gallinoro E Paolisso P Bertolone DT Fabbricatore D. Safety of right and left ventricular endomyocardial biopsy in heart transplantation and cardiomyopathy patients. JACC Heart Failure. (2022) 10(12):963–73. 10.1016/j.jchf.2022.08.005 36456070

[93] Goldberg JF Tian X Bon A Xu Y Gerhard E Brower R. Redefining cardiac antibody-mediated rejection with donor-specific antibodies and graft dysfunction. Circulation Heart Failure. (2024) 17(12). 10.1161/CIRCHEARTFAILURE.124.011592 PMC12147663 39584219

[94] Cusi V Cardenas A Tada Y Vaida F Wettersten N Chak J. Surveillance donor-specific antibody and pathologic antibody-mediated rejection testing in heart transplant patients in the contemporary era. J Heart Lung Transplant. (2025) 44(7):1036–49. 10.1016/j.healun.2025.01.019 39914762 PMC12206481

[95] Singh D. Immunosuppression for postcardiac transplant patients. Journal of the Practice of Cardiovascular Sciences. (2018) 4(3):159.

[96] Crepeau RL Ford ML. Challenges and opportunities in targeting the CD28/CTLA-4 pathway in transplantation and autoimmunity. Expert Opin Biol Ther. (2017) 17(8):1001–12. 10.1080/14712598.2017.1333595 28525959 PMC5590720

[97] Bery AI Belousova N Hachem RR Roux A Kreisel D. Chronic lung allograft dysfunction: clinical manifestations and immunologic mechanisms. Transplantation. (2024) :109. 10.1097/TP.0000000000004962 PMC11799353 39104003

[98] Bos S Milross L Filby AJ Vos R Fisher AJ. Immune processes in the pathogenesis of chronic lung allograft dysfunction: identifying the missing pieces of the puzzle. Eur Respir Rev. (2022) 31(165):220060. 10.1183/16000617.0060-2022 35896274 PMC9724884

[99] Costa J Benvenuto LJ Sonett JR. Long-term outcomes and management of lung transplant recipients. Best Pract Res Clin Anaesthesiol. (2017) 31(2):285–97. 10.1016/j.bpa.2017.05.006 29110800

[100] Olanipekun T Divo M Abe T Taddesse A Poli S Jacob S. Respiratory oscillometry in monitoring lung transplant allograft function: a systematic scoping review. Eur Respir Rev. (2026) 35(179):250018. 10.1183/16000617.0018-2025 41534887 PMC12801049

[101] Fricke K Sievi NA Schmidt FP Schuurmans MM Kohler M. Efficacy of surveillance bronchoscopyversusclinically indicated bronchoscopy for detection of acute lung transplant rejection: a systematic review and meta-analysis. ERJ Open Res. (2024) 10(5):00404–2024. 10.1183/23120541.00404-2024 39377093 PMC11456970

[102] Brun AL Chabi ML Picard C Mellot F Grenier PA. Lung transplantation: CT assessment of chronic lung allograft dysfunction (CLAD). Diagnostics. (2021) 11(5):817. 10.3390/diagnostics11050817 33946544 PMC8147203

[103] Keller M Agbor-Enoh S. Donor-derived cell-free DNA for acute rejection monitoring in heart and lung transplantation. Curr Transplant Rep. (2021) 8(4):351–8. 10.1007/s40472-021-00349-8 34754720 PMC8570240

[104] Thabut G Mal H. Outcomes after lung transplantation. J Thorac Dis. (2017) 9(8):2684–91. 10.21037/jtd.2017.07.85 28932576 PMC5594127

[105] Hayes D Avdimiretz N Brugha R Mullen MP Paraskeva MA Midyat L. International society for heart and lung transplantation consensus statement on the referral and selection of pediatric lung transplant candidates. J Heart Lung Transplant. (2025) 44(12):e133–76. 10.1016/j.healun.2025.08.005 41060377

[106] Hehir CM O’Connor M Marinescu I Dengu F Giele HP Dolan RT. Vascularised composite allotransplantation: emerging applications in reconstructive surgery and solid organ transplantation. Medicina (B Aires). (2026) 62(2):245. 10.3390/medicina62020245 PMC12941565 41752645

[107] Uluer MC Brazio PS Woodall JD Nam AJ Bartlett ST Barth RN. Vascularized composite allotransplantation: medical complications. Curr Transplant Rep. (2016) 3(4):395–403. 10.1007/s40472-016-0113-x 32288984 PMC7101879

[108] Wang W Li W Cao L. Serum extracellular vesicle MicroRNAs as candidate biomarkers for acute rejection in patients subjected to liver transplant. Front Genet. (2022) 13. 10.3389/FGENE.2022.1015049 PMC9606588 36313425

[109] van den Bosch TPP Hilbrands LB Kraaijeveld R. Pretransplant numbers of CD16+ monocytes as a novel biomarker to predict acute rejection after kidney transplantation: a pilot study. Am J Transplant. (2017) 17(10):2659–2667. 10.1111/ajt.14280 28332287

[110] Mathew JM Ansari MJ Gallon L Leventhal JR. Cellular and functional biomarkers of clinical transplant tolerance. Hum Immunol. (2018) 79(5):322–333. 10.1016/J.HUMIMM.2018.01.009 29374560

[111] Kishimoto TK Maldonado RA. Nanoparticles for the induction of antigen-specific immunological tolerance. Front Immunol. (2018) 9. 10.3389/FIMMU.2018.00230 PMC5826312 29515571

[112] Tran J Sharma D Gotlieb N Xu W Bhat M. Application of machine learning in liver transplantation: a review. Hepatol Int. (2022) 16(3):495–508. 10.1007/S12072-021-10291-7 35020154

[113] Jiang L Wang J Wang Y. Bibliometric and LDA analysis of acute rejection in liver transplantation: emerging trends, immunotherapy challenges, and the role of artificial intelligence. Cell Transplant. (2025) 34. 10.1177/09636897251325628 PMC11951891 40152403

