# Correction: Graft rejection across solid organ transplants: mechanisms, monitoring, and immunosuppressive therapeutics

**DOI:** 10.3389/fsurg.2026.1920689

**Published:** 2026-08-03

**Authors:** Edward Akosah Danso, Malik Olatunde Oduoye, Williams Chukwuebuka Enuh, Umer Wamiq, Hafsa Shuja, Fnu Sawaira, Hareem Fatima, Sadia Tameez-ud-din

**Affiliations:** 1 Department of Surgery, Korle-Bu Teaching Hospital, Accra, Ghana; 2 Department of Research, The Medical Research Circle (MedReC), Goma, Democratic Republic of Congo; 3 Department of Health Sciences, Hamburg University of Applied Sciences, Hamburg, Germany; 4 Department of Medicine, Jinnah Sindh Medical University, Karachi, Pakistan; 5 Department of Medicine, Khyber Girls Medical College, Peshawar, Pakistan; 6 Dow University of Health Sciences, Karachi, Pakistan; 7 Department of Medicine, Foundation University Medical College, Islamabad, Pakistan

**Keywords:** biological agents, graft rejection, immune tolerance, immunosuppressive therapy, organ transplantation, transplant outcomes

Author affiliation

Wrong affiliation, but not replacing with present address

Affiliation [Dow University of Health Sciences, Karachi, Pakistan] was erroneously given as [Department of Medicine, Dow University of Health Sciences, Lahore, Pakistan].

The original version of this article has been updated.

Missing citation

Wrong

The reference for [71] was erroneously written as [Wagner-Skacel J, Fink N, Kahn J, et al. Improving adherence to immunosuppression after liver or kidney transplantation in individuals with impairments in personality functioning—A randomized controlled feasibility study. Front Psychol. (2023) 14:1150548. 10.3389/fpsyg.2023.1150548].

It should be [Turolo SE, defonti A,Syren ML,Montini G. Pharmacogenomics of old and new immunosuppressive drugs for precision medicine in kidney transplantation. J Clin Med. (2023) 12(13):4454. 10.3390/JCM12134454].

The reference for [72] was erroneously written as [Turolo S, Edefonti A, Syren ML, Montini G. Pharmacogenomics of old and new immunosuppressive drugs for precision medicine in kidney transplantation. J Clin Med. (2023) 12(13):4454. 10.3390/JCM12134454].

It should be [Saad AF, Pacheco LD, Saade GR. Immunosuppressant Medications in Pregnancy. Obstet Gynecol. (2024) 143(4):e94-e106. 10.1097/AOG.0000000000005512].

The reference for [73] was erroneously written as [Hussain Y, Khan H. Immunosuppressive drugs. Encyclop Infect Immun. (2022) 4:726. 10.1016/B978-0-12-818731-9.00068-9].

It should be [Schonder KS, Mazariegos GV, Weber RJ. Adverse effects of immunosuppression in pediatric solid organ transplantation. Paediatr Drugs. (2010)12(1):35-49. 10.2165/11316180-000000000-00000].

The reference for [74] was erroneously written as Khalid H, Fareed MM, Dandekar T, Shityakov S. Calcineurin and mTOR inhibitors in kidney transplantation: integrative metamodeling on transplant survival and kidney function. Int Urol Nephrol. (2024) 56:1403–1414. 10.1007/s11255-023-03987-9].

It should be [Zicarelli M, Errante A, Andreucci M, Coppolino G, Bolignano D. Immunosuppressive Therapy, Puberty and Growth Outcomes in Pediatric Kidney Transplant Recipients: A Pragmatic Review. Pediatr Transplant. (2024) 28(7):e14878. 10.1111/petr.14878].

The reference for [75] was erroneously written as [Storek J, Lindsay J. Rituximab for post-transplant lymphoproliferative disorder: therapeutic, preemptive, or prophylactic? Bone Marrow Transplant. (2023) 58(7):789–798. 10.1038/s41409-023-01987-2].

It should be [Miloh T, Barton A, Wheeler J, et al. Immunosuppression in pediatric liver transplant recipients: Unique aspects. Liver Transpl.(2017) 23(2):244-256. 10.1002/lt.24677].

The reference for [76] was erroneously written as [Mitsunaga S, Yamada Y, Nguyen PT, et al. A SNP-based capture and clustering workflow to assess donor-derived cell-free DNA in transplantation. PLoS One. (2026) 21(2):e0342082. 10.1371/journal.pone.0342082].

It should be [Krenzien F, El Hajj S, Tullius SG, Gabardi S. Immunosenescence and Immunosuppressive Drugs in the Elderly. Handbook of Immunosenescence. (2019) pp 2147–2167. 10.1007/978-3-319-99375-1_137].

The original version of this article has been updated.

Text correction

Adding/removing text

An error was made in interpretation of the references. Moreover, the word Pharmacogenetic has been changed to Pharmacogenomics, since the idea of polymorphic genes was discussed. The original text is as follows:

Adverse effects also impair quality of life (70) and contribute to non-adherence, reported in 20%–70% of transplant patients (69). Non-adherence increases the risk of acute rejection, graft failure, and antibody-mediated rejection, with younger patients particularly vulnerable (70). Sociodemographic, treatment-related, and psychological factors further exacerbate noncompliance (71). Pharmacogenetic testing offers potential to tailor regimens and reduce toxicity (72), though it is rarely implemented in routine practice (72).

Special populations present additional challenges. Altered pharmacokinetics, immunosenescence, infection risk, and drug interactions complicate therapy in pregnant, pediatric, elderly, and organ-impaired patients (73–75). Pregnancy and breastfeeding are generally contraindicated due to teratogenicity and drug secretion in breast milk (73, 74, 76), though data on infant risk remain limited (51, 53, 70). Elderly patients face higher risks due to comorbidities and reduced clearance (51, 74), while children encounter issues of growth suppression and limited safety data (23, 51, 53, 56, 70, 76). These multifaceted challenges underscore the complexity of balancing efficacy, toxicity, and long-term safety in immunosuppressive management.

A correction has been made to the section [Section 3: Results, Subsection 3.6: Challenges in Immunosuppressive therapy, paragraph 2 and 3]:

“Adverse effects also impair quality of life (70) and contribute to non-adherence, reported in 20%–70% of transplant patients (69). Non-adherence increases the risk of acute rejection, graft failure, and antibody-mediated rejection, with younger patients particularly vulnerable (70). Sociodemographic, treatment-related, and psychological factors further exacerbate noncompliance (69). Pharmacogenomics offers potential to tailor regimens and reduce toxicity (71), though it is rarely implemented in routine practice (71).

Special populations present additional challenges. Most immunosuppressants are safe during pregnancy and lactation, some require monitoring and some are strictly contraindicated (72). As addressed in the reviews, pediatric patients face challenges in development and quality of life (73, 74, 75). Whereas, senescence-related changes in pharmacokinetics and comorbidities pose challenges for geriatric patients (76). These multifaceted challenges underscore the complexity of balancing efficacy, toxicity, and long-term safety in immunosuppressive management.”

The original version of this article has been updated.

