# Supplementary material for: Graft rejection across solid organ transplants: mechanisms, monitoring, and immunosuppressive therapeutics
Source: Front Surg. 2026 Apr 20;13:1762417. doi: 10.3389/fsurg.2026.1762417 (PMC13136141; doi:10.3389/fsurg.2026.1762417)
Supplement: Supplementary file 1 [file Table1.docx]

**Search Strategy:**

**PubMed:**

("Organ Transplantation"[Mesh] OR "organ transplantation" OR "solid organ transplant" OR "kidney transplant" OR "liver transplant" OR "heart transplant" OR "lung transplant") AND ("Graft Rejection"[Mesh] OR "graft rejection" OR "transplant rejection" OR "hyperacute rejection" OR "acute rejection" OR "chronic rejection" OR "antibody mediated rejection" OR "cell mediated rejection") AND ("Immunosuppressive Agents"[Mesh] OR "immunosuppressants" OR "calcineurin inhibitors" OR "tacrolimus" OR "cyclosporine" OR "mycophenolate mofetil" OR "azathioprine" OR "corticosteroid" OR "basiliximab" OR belatacept OR "rituximab" OR "biologic agent")

**Google Scholar:**

("organ transplantation" OR "solid organ transplant") AND ("graft rejection" OR "transplant rejection") AND ("immunosuppressive therapy" OR "calcineurin inhibitors" OR "tacrolimus" OR "cyclosporine" or "corticosteroids")

**Cochrane:**

("organ transplantation" OR "solid organ transplantation") AND ("graft rejection" OR "transplant rejection") AND ("immunosuppressive agents" OR "calcineurin inhibitors" OR tacrolimus OR cyclosporine OR belatacept)

**ClinicalTrials.gov:**

(graft rejection OR transplant rejection) AND (immunosuppressive therapy OR biologic agents OR calcineurin inhibitors)
